# Characterization of Recombinant His-Tag Protein Immobilized onto Functionalized Gold Nanoparticles

**DOI:** 10.3390/s18124262

**Published:** 2018-12-04

**Authors:** Lisa Torres-González, Ramonita Díaz-Ayala, Carmen A. Vega-Olivencia, Juan López-Garriga

**Affiliations:** Department of Chemistry, University of Puerto Rico-Mayagüez Campus, Mayagüez 00680, Puerto Rico; lisa.torres1@upr.edu (L.T.-G.); ramonita.diaz@upr.edu (R.D.-A.); carmenamaralis.vega@upr.edu (C.A.V.-O.)

**Keywords:** protein immobilization, hydrogen sulfide, X-ray photoelectron spectroscopy, *Lucina pectinata*, His-tag protein

## Abstract

The recombinant polyhistidine-tagged hemoglobin I ((His)_6_-rHbI) from the bivalve *Lucina pectinata* is an ideal biocomponent for a hydrogen sulfide (H_2_S) biosensor due to its high affinity for H_2_S. In this work, we immobilized (His)_6_-rHbI over a surface modified with gold nanoparticles functionalized with 3-mercaptopropionic acid complexed with nickel ion. The attenuated total reflection Fourier transform infrared spectroscopy (ATR-FTIR) analysis of the modified-gold electrode displays amide I and amide II bands characteristic of a primarily α-helix structure verifying the presence of (His)_6_-rHbI on the electrode surface. Also, X-ray photoelectron spectroscopy (XPS) results show a new peak after protein interaction corresponding to nitrogen and a calculated overlayer thickness of 5.3 nm. The functionality of the immobilized hemoprotein was established by direct current potential amperometry, using H_2_S as the analyte, validating its activity after immobilization. The current response to H_2_S concentrations was monitored over time giving a linear relationship from 30 to 700 nM with a corresponding sensitivity of 3.22 × 10^−3^ nA/nM. These results confirm that the analyzed gold nanostructured platform provides an efficient and strong link for polyhistidine-tag protein immobilization over gold and glassy carbon surfaces for a future biosensors development.

## 1. Introduction

Protein immobilization on solid supports has become an essential process to develop biomaterials, including biosensors, and to study protein-protein interactions and analyte-protein interactions [1,2]. The main challenges in protein immobilization are maintaining the protein functionality and controlling the orientation to ensure equal access of the analyte to the active sites. Oriented immobilization is mostly addressed using recombinant fusion proteins [3,4,5] where a polypeptide tail is added to the protein on either side of the N- or C-terminus. With these proteins, an oriented immobilization is achieved using affinity-based methods between the tag and a biocompatible surface. Polyhistidine-tags are popular affinity tags, immobilized via a metal-chelated complex, used in protein purification [6,7] and biosensor development [8]. Most common metal ions include Ni^2+^, Co^2+^, and Cu^2+^ linked to nitrilotriacetic acid (NTA) as a chelating agent.

Self- assembled monolayers (SAMs) of alkanethiolates are a plausible alternative to NTA [9,10,11,12]. SAMs of thiols on nanoparticles have been employed as a platform for the construction of biomaterials. The nanoscale properties, such as high conductivity and biocompatibility, coupled with the high selectivity offered by the biomolecules are ideal for biomaterial development [13]. The most popular nanomaterial to conjugate with thiols are gold nanoparticles (AuNPs) due to their extraordinary optical, electronic, and molecular recognition properties [14,15]. Furthermore, AuNPs provide an appropriate environment for preserving the protein biological activity.

The hemoglobin I (HbI), from the clam *Lucina pectinata* has a unique sequence of amino acids in the active center which are thought to be responsible for the unusually high affinity for hydrogen sulfide (H_2_S) [16,17,18,19,20]. Due to this high affinity for H_2_S, HbI has been proposed as a biocomponent for an H_2_S-biosensor [11,21] and to study H_2_S-protein interactions [19,22]. The native HbI has been cloned with an hexahistidine tag [20] and was recently fused with an hexalysine tag [23]. The recombinant proteins maintained the same structural and binding properties of the native protein. The polyhistidine-tag protein, (His)_6_-rHbI, has shown electrochemical activity to H_2_S after immobilization over a flat gold electrode [11]. Whereas the recombinant polylysine tag protein, (Lys)_6_-rHbI), was immobilized over carbon nanotubes via covalent conjugation [21]. These systems successfully demonstrated protein immobilization and functionality toward H_2_S, however, surface characterization and surface coverage were mostly limited by the chosen surface.

Recently, a recombinant protein of HbI was used for the reversible detection and quantification of H_2_S by fluorescence analysis [24]. The fluorescent system can detect H_2_S in real time with detection limits (LOD) around 200 nM at pH 7.4. Another sensor that measures H_2_S in real time is the polarographic hydrogen sulfide sensor (PHSS) with LOD as low as 10 nM at pH 7 [25]. In PHSS, HbI has been used for calibration by adding a known concentration of the protein to scavenge H_2_S. The high H_2_S-affinity presented by HbI of *L. pectinata* makes it an ideal candidate for an H_2_S biosensor and for the study of protein interactions with different analytes. In this work, we studied the oriented immobilization and characterization of (His)_6_-rHbI over a conductive surface. We explored the biosensing capabilities of the immobilized protein to corroborate protein activity; however, the optimization as an H_2_S-sensor will be explored in future work.

The objective of this work was to explore the use of gold nanoparticles (AuNPs) as a platform, over two different substrates, to immobilize the (His)_6_-rHbI and to improve surface characterization. The system under study consisted on the electrodeposition of gold nanoparticles over a bare surface, i.e., gold (Au) or glassy carbon (GC), to enhance surface properties and to immobilize (His)_6_-rHbI via SAMs of 3-mercaptopropionic acid complexed with nickel ion. Surface analytical characterization, by X-ray photoelectron spectroscopy (XPS), corroborated the presence and orientation of the immobilized (His)_6_-rHbI over AuNPs. Also, attenuated total reflection Fourier transform infrared spectroscopy (ATR-FTIR) analysis showed the characteristic bands of amide I and amide II corresponding to a primarily alpha-helix structure maintaining structural integrity after protein immobilization. Electrochemical amperometry analysis confirmed protein activity by showing a linear response of the immobilized (His)_6_-rHbI to H_2_S within the tested range of 30 to 700 nM.

The results presented in this work suggest that AuNPs provide a suitable environment to immobilize polyhistidine-tagged proteins over the different substrates, and as a platform for the development of electrochemical biosensors.

## 2. Materials and Methods

### 2.1. Recombinant Hemoglobin I Expression and Purification

Recombinant polyhistidine HbI was expressed in *E. coli* and purified per the procedures previously established [20]. Induction was achieved with 0.001 M isopropyl β-d-1-thiogalacto-pyranoside (IPTG) at 30 °C in Terrific Broth (TB) medium supplemented with 30 μg/mL hemin chloride and 1% glucose. The red cell pellets obtained were lysed and centrifuged to separate the soluble protein fraction for further purification. The (His)_6_-rHbI was purified with metal affinity columns and by fast performance liquid chromatography (FPLC) in a HiLoad 26/60 Superdex 200 gel filtration column. After size exclusion chromatography, the protein purity and integrity was corroborated by sodium dodecyl sulfate-polyacrylamide gel electrophoresis (SDS-PAGE) stained with Coomassie blue as an initial sample assessment [26]. The SDS-PAGE shows only one band around 17 kDa which is similar to the molecular weight of (His)_6_-rHbI [20].

### 2.2. Apparatus and Reagents

All reagents were analytical grade and used as received, i.e., 3-mercaptopropionic acid (3-MPA), nickel chloride (NiCl_2_), sodium sulfide (Na_2_S), ethanol (C_2_H_5_OH), sulfuric acid (H_2_SO_4_), chloroauric acid (HAuCl_4_) and sodium nitrate (NaNO_3_). Sodium sulfide salt was dissolved in phosphate buffer (pH 6.5) previously degasified and purged with nitrogen. Deionized water was used throughout.

The XPS studies were carried out on a PHI 5600 ci spectrometer (Physiscal Electronics, Chanhassen, MN, USA) with a standard Al-Kα source at 350.0 W. All spectra were calibrated using the adventitious carbon (C1s) peak at 284.8 eV. The FTIR measurements were done on a Spectrum 100 FT-IR spectrometer (PerkinElmer, San Juan, PR) fitted with a Universal ATR accessory. An Atomic Force Microscopy instrument, an AutoProbe CP, from Park Scientific Instruments (Albany, NY, USA) was used to take images of the nanogold in non-contact mode. The Au substrate used for AFM and XPS analysis was a quartz sensor crystal with a gold-coated thin film.

The electrochemical measurements were performed on a BASi Epsilon Potentiostat-Galvanostat station (Bioanalytical Systems, Inc., West Lafayette, IN, USA) with a three-electrode cell, where gold (Au) was used as the working electrode, and a platinum wire and an Ag/AgCl (3 M NaCl) electrode acted as the counter and reference electrodes, respectively. All electrochemical measurements were made using IUPAC convention. The cyclic voltammetry experiments were run from −200 to 600 mV starting at the open circuit potential at a scan rate of 20 mV/s. The amperometry analysis was done on a stirred cell at room temperature applying a potential of 230 mV vs. Ag/AgCl with a sample interval time of 0.5 s.

### 2.3. Electrode Preparation

**Figure 1:**
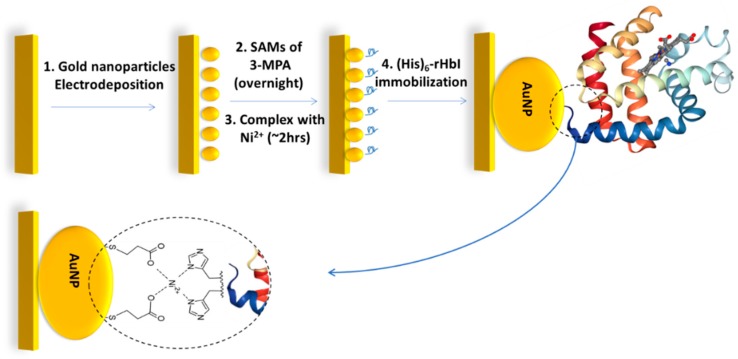
Schematic procedure of gold electrode modification steps for the (His)_6_-rHbI protein immobilization over AuNPs via polyhistidine-tag with 3-MPA/Ni^2+^ complex.

The schematic procedure of electrode modification is described in Figure 1. Before the AuNPs’ electrodeposition, working Au electrodes were polished on a velvet pad with alumina, then sonicated in deionized water for two minutes, and electrochemically cleaned in 0.5 M H_2_SO_4_ by potential scanning between –0.375 and 1.8 V until a reproducible and characteristic cyclic voltammogram for the clean gold electrode was obtained. Electrodeposition of AuNPs over the Au and GC surfaces was achieved using controlled potential electrolysis (CPE) in a solution containing 1.2 mM of HAuCl_4_ with NaNO_3_ as supporting electrolyte, for 30 to 60 s with an applied potential of 0.0 V, which implies an overpotential of around −0.8 V vs. Ag/AgCl, according to the following reaction: HAuCl_4_ + 3e^−^ → Au + 4HCl (E° = 1.00 V vs. SHE).

**Figure 2:**
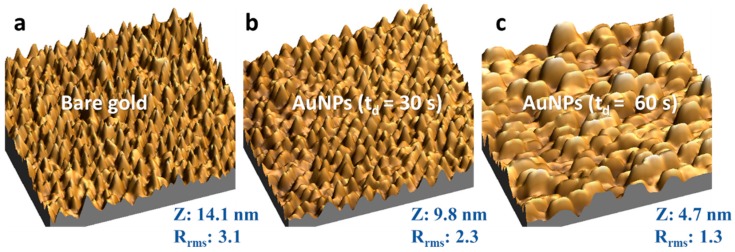
Three-dimensional AFM images (1 μm × 1 μm) of the gold electrode before and after AuNPs electrodeposition by controlled potential electrolysis with 0.0 V applied potential in a solution of 0.25 mM HAuCl_4_ with NaNO_3_. The Z corresponds to the average height, and R_rms_ is the root mean square average roughness factor of each sample. (**a**) bare gold electrode; (**b**) after AuNPs electrodeposition with td = 30 s; (**c**) after AuNPs electrodeposition with td = 60 s.

Figure 2 shows the AFM images (1 μm × 1 μm) of AuNPs over the gold substrate. The time-dependent size of AuNPs is in the range of 20–150 nm of extent with gold nano-island formation instead of spherical nanoparticles. The z-height decrease as the electrodeposition time increases since the electrodeposited AuNPs fill the valleys of the bare electrode resulting in decreased height and smoother surfaces, as revealed by the root mean square roughness factor (R_rms_).

The nanostructured electrode was left overnight in 3-MPA ethanolic solution to form thiol-SAMs. After rinsing the electrode, it was placed in a NiCl_2_ solution for approximately two hours to obtain the modification with the 3-MPA/Ni^2+^ complex (Au/AuNPs/3MPA/Ni^2+^) to be used as the platform to immobilize (His)_6_-rHbI. To immobilize (His)_6_-rHbI on SAMs, the modified electrode was left in the protein solution overnight. After the last modification step, the electrodes were rinsed with copious amounts of deionized water and dried with nitrogen gas for further analysis.

### 2.4. Data Analysis Software

AFM images were processed and analyzed using the Scanning Probe Microscopy software WSxM 5.0 Develop 8.4 (Centro Empresarial Euronova, Madrid, Spain) [27]. ATR-FTIR data collection and processing was achieved with PerkinElmer’s Spectrum FTIR software. Electrochemical results such as cyclic voltammetry (CV) and direct current potential amperometry (DCPA) were processed with the BASI ComServer Version 1.03 Firmware. XPS data analysis was performed using MultiPak software Version 9.4.0.7 from Ulvac-phi, Inc. (Kanagawa, Japan). The atomic percentages were calculated from the experimentally determined peak intensities. Peak fitting was done with no smoothing using symmetric Gaussian-Lorentzian (around 70 and 30 percent respectively) functions after a Shirley-type background subtraction. NIST Standard Reference Database 82 Version 1.3 [28] was used to calculate the electron effective-attenuation-length (EAL) with an electron kinetic energy of 1486.6 eV from Al X-ray source at 350.0 W. For this calculation we assumed a uniform, organic overlayer of protein character with an average density of 1.45 g/cm^3^. This protein density was calculated with the exponential function described by Fischer [29], using the molecular weight of (His)_6_-rHbI obtained from its amino acid sequence [20]. The stoichiometric coefficients of (His)_6_-rHbI are 0.64 C, 0.18 N and 0.19 O; and the number of valence electrons per molecule (Nv = 4.6) was calculated from the sum of contributions from each constituent element as suggested in SRD-82 guide [28]. A band gap energy of Eg = 4.3 eV was used, which corresponds to the typical absorption peak of amino acids in proteins at 280 nm.

## 3. Results and Discussion

### 3.1. Evaluation of (His)_*6*_-rHbI Protein Secondary Structure after Immobilization

**Figure 3:**
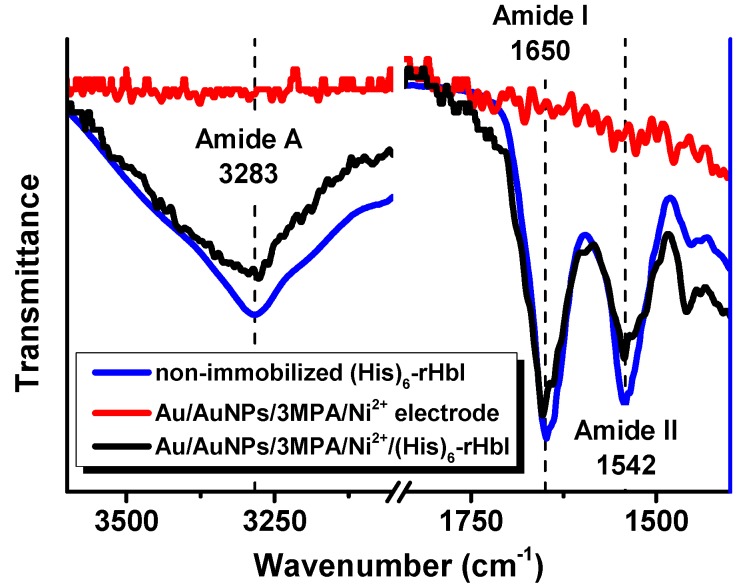
ATR-FTIR spectra, in amide A, and amide I and amide II regions, of non-immobilized (His)_6_-rHbI (blue line), electrode before protein immobilization, AuNPs/3MPA/Ni^2+^ (red line), and electrode after protein immobilization, AuNPs/3MPA/Ni^2+^/(His)_6_-rHbI (black line).

The ATR-FTIR technique was used to corroborate the presence of the protein, and the secondary structural conformation after immobilization. We compare the IR-spectra in the amide A, amide I and amide II regions of the non-immobilized protein with the immobilized (His)_6_-rHbI protein over gold. The amide A band is mainly due to N-H stretching vibration in resonance with amide II, while amide I absorptions result primarily from the C=O stretching vibration of the amide group, and amide II absorptions arise from N-H bending and C–N stretching vibrations [30,31,32]. The amide I and amide II band locations are commonly used to analyze secondary structure changes since they are directly related to the backbone conformation and are hardly affected by the nature of the side chain [33]. The sensitivity of the amide I vibration to secondary structure makes it possible to study protein conformational changes upon adsorption on solid substrates. Figure 3 illustrates the IR spectra of the amide A, amide I and amide II regions for the (His)_6_-rHbI in direct contact with the diamond of the instrument (blue line), the IR of the electrode before the (His)_6_-rHbI immobilization (red line), and the immobilized protein over the modified gold electrode (black line). The IR-spectra of the non-immobilized protein concurs with the spectra of the immobilized protein in those regions, showing bands at 3283 cm^−1^ in the amide A region and 1650 cm^−1^ and 1542 cm^−1^ corresponding to amide I and amide II. There is a slight shift to higher wavenumbers of the amide I and amide II bands, of approximately 4 cm^−1^, relative to the values of the non-immobilized (His)_6_-rHbI protein probably due to Au-surface interaction.

These wavenumbers of amide I and amide II agree with the typical values for proteins with primarily α-helical structures [28], consistent with the crystallographic structure of the native HbI protein [34]. Previous analyses of small/wide angle X-ray scattering (SWAXS) show that the native HbI protein is similar in global three-dimensional structure to the (His)_6_-rHbI protein [19,23]. In addition, before the adsorption of the protein, the spectrum of the Au/AuNPs/3MPA/Ni^2+^ electrode (Figure 3, red line) did not show the characteristic IR bands of proteins in the amide regions. These observations support successful protein immobilization without suffering significant changes in conformation. However, to corroborate protein functionality, an electrochemical analysis was performed and will be discussed in the Electrochemical Analysis, Section 3.5.

### 3.2. XPS Analysis of the Gold Electrode in Each Step of the Surface Modification

**Figure 4:**
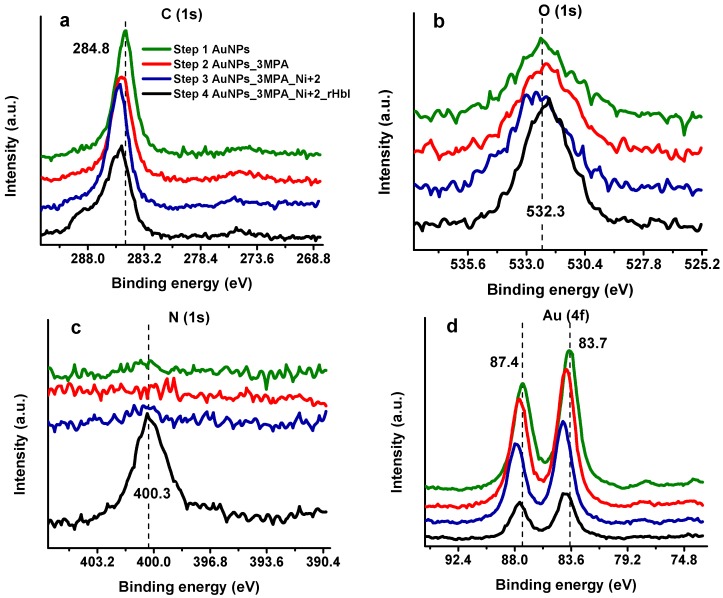
High-resolution XPS spectra of gold electrode consecutive modification steps in (**a**) C 1s region, (**b**) O 1s region, (**c**) N 1s region, (**d**) Au 4f region. The modification steps are: (1) AuNPs electrodeposition (green line) (2) 3-MPA SAMs (red line) (3) Ni^2+^ complex with 3-MPA (blue line) (4) (His)_6_-rHbI immobilization step (black line).

X-ray photoelectron spectroscopy has been applied recently to characterize and quantify the protein layer over AuNPs, allowing the detection of the protein and the determination of the surface coverage over the substrate [35,36,37]. Moreover, XPS provides accurate information about the chemical environment and how it changes as the electrode is modified. Figure 4 compares XPS high-resolution spectra (HRS) at regions of carbon (C 1s), nitrogen (N 1s), oxygen (O 1s), and gold (Au 4f) in the consecutive steps of the modification process: (1) Bare gold electrode with electrodeposited AuNPs, (2) AuNPs with SAMs of 3-MPA, (3) AuNPs/3MPA complexed with Ni^2+^, and (4) AuNPs/3MPA/Ni^2+^ with immobilized (His)_6_-rHbI protein. Each modification step shows differences between XPS spectra suggesting changes in the chemical environment at the surface, especially after protein adsorption. In the C 1s region of the first modification step, presented in Figure 4a (green line), the carbon peak is attributed to adventitious carbon contamination (C–C, C–H at 284.8 eV) which is commonly found in all samples exposed to the atmosphere. The next two steps in Figure 4a correspond to SAMs of 3-MPA over AuNPs (red line) and to the AuNPs/3-MPA/Ni^2+^ (blue line) modifications. Compared to the previous step, there is displacement to higher binding energies (BE). This shift is evidence of a new chemical environment probably corresponding to the S–C (~286.5 eV) and O–C=O (~288.5 eV) present in the 3-MPA molecule, an indication of successful SAMs formation. Additionally, in the last step of the modification process when the protein is adsorbed (Figure 4a, black line), the spectrum shows a broader peak with a bump to higher BE indicating an increase in the concentration of new species of carbon, related to amine and amide groups coming from the immobilized protein. The XPS results in the last modification step will be further discussed in the peak fitting analysis, Section 3.3.

Figure 4b displays the HRS for the O 1s band. There is a slight shift to higher BE in step 3 (blue line). This chemical shift can be attributed to the highly positively charged Ni^2+^ ion which decreases the electron density (increases BE) of the photoelectrons of oxygen from the carboxylic acid of 3-MPA. However, with the protein on the surface, the oxygen peak has higher intensity, but less broadening than the previous surface modifications. This peak difference is reasonable since most of the electrons reaching the XPS-detector come from the protein, leaving out a greater variety of oxygen electrons from the stepwise modified surface. As can be seen in Figure 4c (black line) a new intense signal appears in the last step of the modification at 400 eV corresponding to the N 1s core-level spectra of the adsorbed protein. These results suggest a successful stepwise surface modification including the adsorption of the (His)_6_-rHbI.

Protein detection and quantification are typically achieved by monitoring changes in the nitrogen content and the surface signal attenuation. In our case, the N 1s signal confirms the presence of (His)_6_-rHbI over the gold modified electrode. At the same time, attenuation of the gold substrate is observed in Figure 4d being more perceptible once the bulky protein is over the surface. The presence of the protein over the AuNPs surface leads to 68% signal attenuation of Au 4f, i.e., the difference between the first (green line) and last (black line) signal areas relative to the first (green line) as shown in Figure 4d. Also, the chemical shift of Au 4f spectra occurs to higher binding energies due to electronegativity effects consistent with Au-S bond formation (Figure 4d, red line) and is even more evident with the presence of 3-MPA/Ni^2+^ complex (Figure 4d, blue line).

### 3.3. Different Chemical Species of C 1s, N 1s, and O 1s Bands after (His)_*6*_-rHbI Adsorption

**Figure 5:**
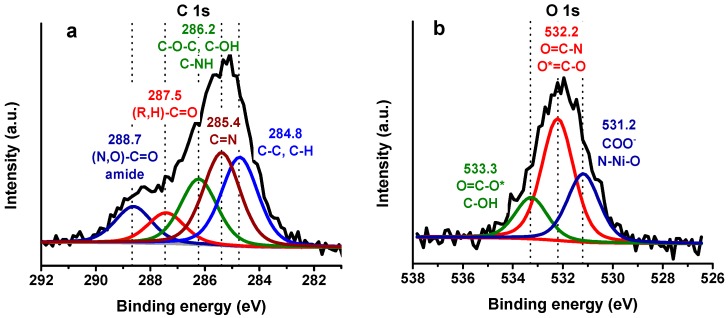

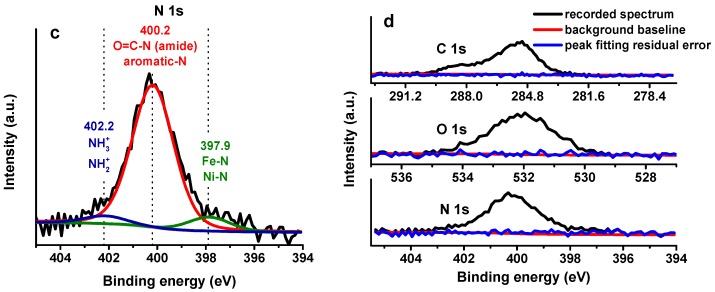
XPS-HRS and peak fitting of (**a**) C 1s, (**b**) O 1s where * refer to the O peak assigned and (**c**) N 1s in the last step of the modification (Au/AuNPs/3MPA/Ni^2+^/(His)_6_-rHbI) with possible peak assignment, (**d**) residual error after peak fitting for C 1s, O 1s, and N 1s.

Peak fitting of XPS-HRS is useful to identify different chemical species present in the electrode surface. Figure 5 illustrates the peak fitting for the last modification step of the most prominent elements found in proteins (C 1s, N 1s, and O 1s). The full width at half maximum (FWHM) for each element were constrained to have the same value. The C 1s spectrum (FWHM ~1.7 eV) was fitted with five components that agreed with proteins bound to a surface, as seen in Figure 5a. The peak at 284.8 eV was attributed to C–C and C–H while the peaks at 285.4 and 286.2 eV were assigned to C–N and C–OH, respectively. The peak at 287.5 eV is related to carbonyl groups of ketonic or aldehydic carbon ((R,H)–C=O). Amide (N–C=O) and carboxylate (O=C–OH) carbon show peaks to higher BE around 288.7 eV. Carbon peak fitting assignments indicate the presence of peptide bonds which are consistent with protein adsorption.

The HRS of O 1s shown in Figure 5b (FWHM ~1.6 eV) was fitted with three distinct peaks at 531.2, 532.2 and 533.3 eV ascribed mainly to COO^−^, O=C–N, and O=C–O, respectively. Likewise, the N 1s core level spectrum (FWHM ~1.7 eV), displayed in Figure 5c, was fitted with three peaks at 397.9, 400.2 and 402.2 eV. The first peak is assigned to the metal-N coordination bond between the porphyrin iron and nitrogen, and to the nickel ion with the N-imidazole from the polyhistidine-tag. In addition, the peak at 400.2 eV can be assigned to the amide-N, aromatic-N, and amine-N of the protein. The peak at 402.2 eV is probably due to the positively charged ammonium in lysine. 

Figure 5d demonstrates the residual error (blue line) for each element analyzed after peak fitting. The chi-squared peak fitting parameter ranges from 1 to 1.5. In summary, the XPS results demonstrated the presence of peptide bonds in all the regions analyzed in Figure 5, verifying protein adsorption over the modified gold electrode. It was also possible to detect the Ni^2+^-N bond in the N 1s region, evidencing protein orientation.

### 3.4. Overlayer Thickness after (His)_*6*_-rHbI Immobilization on Modified Electrode

The attenuation of the XPS substrate signal or the introduction of nitrogen by the adsorption of a protein can be used to calculate the adsorbed film thickness of the surface [36,37,38,39]. The overlayer thickness was calculated by analyzing two samples with the same modification (Au/AuNPs/3MPA/Ni^2+^/(His)_6_-rHbI). The relative overlayer thickness was determined from the attenuation of the Au 4f_7/2_ peak using the equation (1) for normal emission:

I_Au_ = I°_Au_ exp(−t/L_Au_) (1)

where t represents overlayer thickness, L_Au_ is the average practical EAL (effective attenuation length), and I°_Au_ and I_Au_ are the peak intensities (areas) of non-attenuated and attenuated signals respectively. The I°_Au_ corresponds to the Au 4f_7/2_ peak area of the gold sample with electrodeposited AuNPs (Figure 4d, green line), while I_Au_ corresponds to the Au 4f_7/2_ intensity of the sample after protein adsorption (Figure 4d, black line). The EAL was calculated using the NIST Electron Attenuation Database 82 within the predictive formulation TPP-2M [28]. The EAL obtained (L_Au_) was 3.887 nm which resulted, from Equation (1), in an average overlayer thickness of 5.3 nm in the last step of the modification, as shown in Table 1. This thickness corresponds to all layers made over AuNPs, i.e., to 3-MPA/Ni^2+^/(His)_6_-rHbI. The 3-MPA/Ni^2+^ complex is a small molecule compared to (His)_6_-rHbI; thus the thickness comes mostly from the protein film. Table 1 displays the average relative intensities obtained from two samples and the calculated thickness in each stepwise electrode modification.

According to the experimental data in the RSCB Protein Data Bank (PDB id:1B0B), a unit cell of native HbI is 4.944 nm × 3.795 nm × 4.137 nm, which is very similar to our protein thickness results when the thickness of the previous steps is subtracted (~4.7 nm). As mentioned previously, according to SWAXS results published before [19,23], the native HbI protein is similar, in global three-dimensional structure to the (His)_6_-rHbI protein. Furthermore, the SWAXS analysis reported a radius of gyration (Rg) between 1.8–1.9 nm for (His)_6_-rHbI, which corresponds to an average diameter of 3.7 nm. Considering that the calculated overlayer thickness (~5.3 nm) includes the linker between AuNPS and the protein, the XPS results suggest a monolayer of (His)_6_-rHbI over AuNPs substrate.

The XPS average elemental composition percentages obtained for C, O, and N were 66.1, 16.1 and 14.5, respectively. These atomic percent compositions agree with typical theoretical and experimental values found in adsorbed proteins [40]. Table 2 lists the XPS relative atomic ratios, for each sample, and the average obtained when the protein is adsorbed on the surface. These atomic ratios are comparable to the relative atomic composition of (His)_6_-rHbI. The elemental atomic ratios were calculated counting each element (C, O, and N) from the crystallographic data of native HbI and from the sequence of amino acids of the histidine tail in the (His)_6_-rHbI presented by Leon [20]. As expected, the ratios that include the atomic carbon percent, are higher from XPS results than those obtained from the sequence of amino acids. This apparent discrepancy is reasonable since the average depth of analysis for an XPS measurement is approximately from 5 to 10 nm, and we estimated a protein film of (His)_6_-rHbI to be about 4.7 nm. Thus, in the XPS case, carbon photoelectrons from the prior modification (with 3-MPA) are counted, in addition to the photoelectrons coming from the protein. However, the similarity in composition between the two experimental samples and the rHbI sequence suggests that the XPS signals in the last step of the modification are primarily from the immobilized (His)_6_-rHbI over the AuNPs surface.

### 3.5. Electrochemical Analysis of Modified Electrode with (His)_*6*_-rHbI Protein

**Figure 6:**
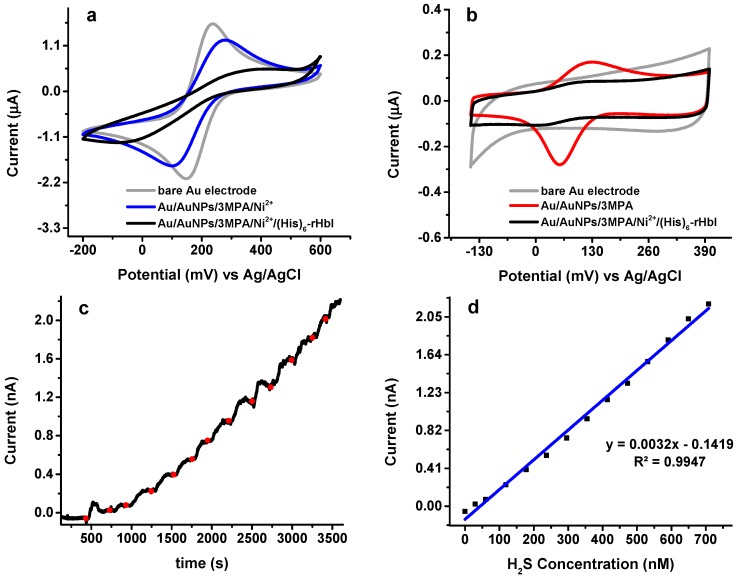
Electrochemical analysis: (**a**) CV in 1.0 mM K_3_Fe(CN)6/0.1 M KCl at 20 mV/s; (**b**) CV in phosphate buffer, pH = 7 at 20 mV/s; (**c**) direct current potential amperometry under an operating potential of 230 mV in phosphate buffer pH = 6.5 with Au/AuNPs/3MPA/Ni^2+^/rHbI electrode; (**d**) corresponding calibration curve of amperometric response toward H_2_S.

In some cases, depending on the immobilization technique used and the selected surface, protein adsorption may lead to loss of protein activity due to random orientation and structural deformation. Electrochemical analysis, shown in Figure 6, is useful to study protein adsorption and to investigate the functionality of the immobilized protein. The (His)_6_-rHbI was immobilized in a standard working gold electrode with electrodeposited AuNPs, following the same procedure that was used for the samples of XPS and FTIR analysis.

Surface modifications were evaluated by CV in a 1.0 mM ferricyanide solution (K_3_Fe(CN)_6_/0.1 M KCl) as illustrated in Figure 6a. The CV of the bare Au electrode (gray line) was compared with the modified electrode before (blue line) and after (black line) protein immobilization. When the electrode is modified with AuNPs/3MPa/Ni^2+^ (blue line) the electron transfer between the ferricyanide solution and the modified electrode shows a current decrease and a potential displacement indicating a surface modification. Moreover, once the protein is adsorbed (black line), the redox peaks are not as perceptible evidencing protein immobilization over the electrode. Similar behavior is observed when the CV is done in phosphate buffer at pH 7, as shown in Figure 6b. In either case, ferricyanide or phosphate buffer, the (His)_6_-rHbI modified electrode presents a significant current reduction in the redox peaks due to the adsorbed protein blocking the electron transfer between the surface and the solution. It is worth mentioning that the Au/AuNPs/3MPA electrode (Figure 6b, red line) exhibits one-pair of peaks corresponding to one electron transfer due to protonation/deprotonation of 3-MPA. The voltammograms of 3-MPA exhibit two pairs of peaks, corresponding to two electron transfers [41], but since thiol groups are strongly adsorbed onto gold through covalent bonds, SAMs of 3-MPA will have high stability and the formation of disulfides is inhibited, resulting in only one electron transfer.

To verify activity of (His)_6_-rHbI after immobilization, DCPA analysis with an applied potential of 230 mV was done with the Au/AuNPs/3MPA/Ni^2+^/(His)_6_-rHbI electrode vs. Ag/AgCl electrode under successive additions of 0.50 to 1.00 μL of Na_2_S aliquots to 20.00 mL of phosphate buffer with pH of 6.5, as seen in Figure 6c. The Na_2_S salt dissolves in the buffer and liberates H_2_S, which bonds to the porphyrin-iron in (His)_6_-rHbI. A steady-state current was reached before each Na_2_S-addition. The average response time of the protein modified electrode to H_2_S was about 30 s which is consistent with other protein-modified electrodes reported in the literature [42]. The H_2_S concentrations were calculated based on the aqueous equilibrium of H_2_S with its ions HS^−^ + S^2−^. Equation (2) was used to calculate the corresponding H_2_S concentration in the equilibrium system:

[H_2_S] = [Na_2_S]/{1 + (K_1_/[H^+^]) + (K_1_K_2_/[H^+^]^2^)} (2)

where pK_1_~pK_7_ and pK_2_~pK_19_. Since the pH of the solution is less than the pK_1_, the equilibrium is shifted toward the formation of H_2_S. Amperometry analysis gives a linear relationship from 30 to 700 nM of H_2_S concentration, as shown in Figure 6d, with a corresponding sensitivity of 3.22 × 10^−3^ nA/nM and a correlation coefficient of 0.995.

The electrochemical analysis showed a current increase when H_2_S was added to the protein-modified electrode which verifies the functionality of the immobilized protein. In addition, the reported LOD falls within the physiologically relevant concentrations of hydrogen sulfide as reported in the literature [43,44,45], ranging from nM to low μM depending on the biological sample and the measuring method used.

As mentioned before, our group published the adsorption of (His)_6_-rHbI over a flat gold surface and established electrochemical activity of the protein to H_2_S after immobilization. The linear response for that electrode ranged from 40 nM to 600 nM, and the lower limit of quantification (LOQ) was 43 nM [11]. However, in the XPS analysis of that modification, it was not possible to detect the presence of nitrogen, N(histidine)-Ni^2+^ nor the presence of the iron-porphyrin bond, probably due to low protein surface coverage. In our work, with the introduction of AuNPs, we were able to improve surface characterization by detecting nitrogen, including species of (Fe, Ni^2+^)-N, after protein adsorption, as seen in Figure 5c. On the other hand, the immobilization over a flat gold electrode obtained a higher electrochemical sensitivity (0.1166 nA/nM), than the immobilization over AuNPs (0.00322 nA/nM). This contrast in sensitivity could also be attributed to differences in the protein surface coverage and the electroactive area between the two bioelectrodes. Although the focus of this work is not the electrochemical optimization of an H_2_S-biosensor, the sensitivity of this new approach to immobilize (His)_6_-rHbI is within the sensitivity range of other electrochemical sensors [46]. Table 3 compares the performance characteristics of the commercial PHHS with the two (His)_6_-rHbI based bioelectrodes. The bioelectrode presented in this work has a comparable limit of quantification and a broader range of H_2_S-linear response, when compared to the previous protein immobilization over a flat gold electrode (Au/(His)_6_-rHbI).

### 3.6. XPS Analysis of Glassy Carbon Modification with AuNPs for (His)_*6*_-rHbI Immobilization

Another advantage of AuNPs is that they can be electrodeposited over any conductive surface like, for example, a glassy carbon electrode (GC) or over transparent fluoride-tin oxide (FTO) surface. 

**Figure 7:**
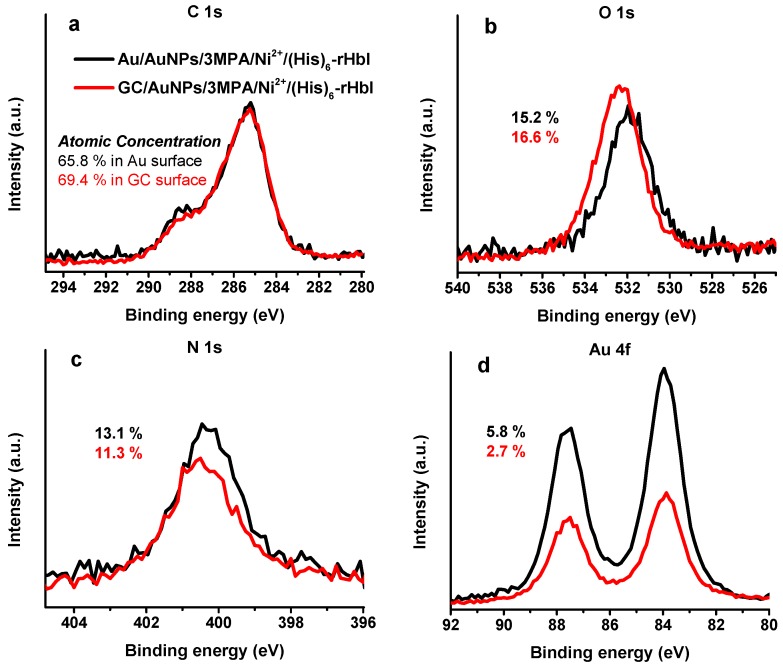
XPS-HRS of glassy carbon (red line) and gold (black line) surfaces modified with AuNPs/3MPA/Ni^2+^/(His)_6_-rHbI using Mg Kα X-ray source of (**a**) C 1s, (**b**) O 1s, (**c**) N 1s, and (**d**) Au 4f regions.

The Au and GC surfaces were modified following the same stepwise modification described in Figure 1, i.e., AuNPs/3MPA/Ni^2+^/(His)_6_-rHbI. Both samples were analyzed by XPS with an Mg Kα X-ray source. Figure 7 shows the HRS obtained for the C 1s, O 1s, N 1s, and Au 4f regions. In the C 1s region (Figure 7a), both spectra are very similar showing a bump to higher BE indicative of new chemical species of carbon, such as amide/peptide bonds, relative to the previous modification step (not shown). For the O 1s region shown in Figure 7b, the GC modification is shifted to higher BE relative to the AuNPs-protein modification. This behavior could be attributed to differences in the surface. Both samples show a prominent nitrogen peak, display in Figure 7c, evidence of the immobilized protein over the modified surface with higher protein concentration in the Au surface. Figure 7d, the Au 4f region, shows the successful electrodeposition of AuNPs over GC surface, and higher intensity for the Au surface, as expected. In the GC sample, the intensity of Au 4f peak was higher after protein immobilization compared to the previous modification step probably due to enlargement of AuNPs. This phenomenon has been observed before and used for the detection of flavonoids [48,49]. The similarity of XPS results of Au and GC surfaces validates that GC, which is more affordable than Au, can also be used as a platform for protein immobilization over AuNPs.

## 4. Conclusions

In summary, the FTIR and XPS results demonstrated the successful and oriented immobilization of (His)_6_-rHbI over AuNPs. Protein activity after adsorption was corroborated by amperometry analysis suggesting the potential use of the (His)_6_-rHbI as the biocomponent for an electrochemical H_2_S-biosensor and to study other analyte-protein interactions. Also, the electrodeposition of AuNPs and the (His)_6_-rHbI immobilization over gold and glassy carbon electrodes proved the versatility of AuNPs as a platform in protein immobilization. In general, the use of AuNPs to immobilize (His)_6_-rHbI improved surface characterization when compared to the previous work reported by our group. As a future work, AuNPs electrodeposition over a transparent FTO surface will allow the spectroelectrochemical analysis of (His)_6_-rHbI interacting with H_2_S and other analytes of interest.

## Figures and Tables

**Table 1:** Attenuation of Gold Substrate Peak Intensities and Overlayer thickness t in each modification step.

Modification Step	Average I_Au_	Average Thickness, t (nm)	Standard Deviation (*n* = 2)
Au/AuNPs	I°_Au_	-	-
Au/AuNPs/3MPA/	51,626	0.11	0.02
Au/AuNPs/3MPA/Ni^2+^	45,229	0.6	0.3
Au/AuNPs/3MPA/Ni^2+^/rHbI	13,476	5.3	1.6

**Table 2:** The relative ratio from XPS atomic concentration percent of two samples and the atomic relative ratio of the (His)_6_-rHbI calculated from the amino acids sequence [20].

Relative Atomic Ratio	XPS Atomic Ratio Percent Sample 1	XPS Atomic Ratio Percent Sample 2	Average XPS Atomic Ratio Percent	Relative Atomic Ratio Percent of (His)_6_-rHbI
O:C	23.0	25.5	24.4	29.1
N:C	21.5	22.3	21.9	27.9
N:O	93.5	87.7	90.1	96.1

**Table 3:** Performance characteristic of H_2_S-electrochemical sensors.

Sensor	Sensitivity (nA/nM)	Detection Limits (nM)	LOQ (nM)	LOD (nM)	pH	Reference
PHSS	0.0055	10 nM to 200 μM	-	10	6	[25,47]
Au/(His)_6_-rHbI	0.1166	40 to 600	43	13	6.7	[11]
AuNPs/(His)_6_-rHbI	0.00322	30 to 700	57	17	6.5	this work
